# Polarization-Selective Bidirectional Absorption Based on a Bilayer Plasmonic Metasurface

**DOI:** 10.3390/ma13225298

**Published:** 2020-11-23

**Authors:** Tong Li, Bin-Quan Chen, Qian He, Li-An Bian, Xiong-Jun Shang, Guo-Feng Song

**Affiliations:** 1 Hunan Provincial Key Laboratory of Flexible Electronic Materials Genome Engineering, Changsha University of Science and Technology, Changsha 410004, China; lt@csust.edu.cn (T.L.); BinquanChen2020@163.com (B.-Q.C.); hqian@csust.edu.cn (Q.H.); dk061bianlian@126.com (L.-A.B.); 2 Institute of Semiconductors, Chinese Academy of Sciences, Beijing 100083, China; sgf@semi.ac.cn; 3 College of Materials Science and Opto-Electronic Technology, University of Chinese Academy of Sciences, Beijing 100049, China; 4 Beijing Key Laboratory of Inorganic Stretchable and Flexible Information Technology, Beijing 100083, China

**Keywords:** polarization-selective, absorb, surface plasmons, metasurface

## Abstract

We propose an alignment-free and polarization-selective bidirectional absorber composed of a one-dimensional bilayer Au grating array buried in a silicon nitride spacer. The absorptivity of the designed structure is more than 95% (77%) under normal forward (backward) TM-polarized light incidence, and is more than 80% (70%) within a forward (backward) incident angle up to 30°. The great bidirectional absorption performance is illustrated by the resonance coupling of the surface plasmon polaritons (SPPs) resonance, the propagating surface plasmon (PSP) resonance and the localized surface plasmon (LSP) resonance under TM-polarized wave illumination. Moreover, the excitation of the Fano-like resonance mode of the proposed metasurface can produce two significantly different peaks in the absorption spectrum under the oblique TM-polarized incidence, which is beneficial for the plasmon-sensing application. Therefore, the proposed bidirectional metasurface absorber can be a candidate in the application of optical camouflage, thermal radiation, solar cells and optical sensing.

## 1. Introduction

Metasufaces, as extraordinary artificial structures, have attracted wide attention due to their outstanding nature in the context of many applications, such as electromagnetic wave stealth [1,2,3], imaging [4,5,6], flat metalens [7,8], optical filters [9], detectors and sensors [10,11]. Compared to the inherent bulk and loss of conventional optical materials, metasurfaces have unparalleled advantages in the integration and miniaturization of optical systems. However, metasurfaces still inevitably undergo some energy losses in their application. Optical absorbers can take the advantage of light loss to create a perfect absorption effect [12,13,14,15,16,17,18]. Perfect optical absorbers without the ability to reflect and transmit light have been the focus interested study for many years, due to their excellent absorption performance. Researchers concentrate their work on theoretical and experimental studies of optical absorbers with various structures, including gratings, nanoparticles, antenna arrays and multilayer composite structures. Many narrow-band, multiband and broadband metasurface-based absorbers have been investigated, and their operating bands covers from the visible region to the microwave band. They play an indispensable role in both science and practical applications, such as solar cells, thermal emitters, photovoltaic devices and sensing.

Polarization, as one of the most important properties of a light source, has attracted extensive attention from researchers. The polarization state can be detected by the flexible control of light absorption, transmission and reflection. Many metasurface-based absorbers (i.e., dielectric metasurfaces, plasmonic metasurfaces and composite metasurface) have been reported, and some promising applications have been discussed [19,20,21,22,23]. For example, metallic film with nanotrenches can strongly couple with polarized light to induce complete absorption, which applies to applications from ultrafast metallic photocathodes and the generation of laser harmonics, through field concentration, to polarization control and switching [24]. A high polarization sensitivity absorber can be used to enhance the contrast detection technology of the target, making up for the lack of intensity detection under complex camouflage conditions [19]. The latest report presents an active tunable polarization-sensitive multiband absorber based on graphene, which has potential applications in mid-infrared polarization-sensitive filters and detectors [25]. However, the designed polarization-selective structure can only absorb light waves from one direction, while reflecting light waves from another direction, which limits the practical application of the device. It is foreseeable that the realization of the bidirectional polarization-selective absorption of light waves can further expand the application of metasurface-based complete absorbers in optical detection.

In this paper, we theoretically demonstrate an alignment-free and polarization-selective bidirectional absorber composed of bilayer gratings buried in a silicon nitride spacer. The simulation results indicate that the absorptivity of the designed device is over 95% (77%), around 666 nm for normal forward (backward) TM-polarized incident light, and can be kept above 80% (70%) within a forward (backward) incident angle up to 30°. The great bidirectional absorption performance is caused by the resonance coupling of surface plasmon polaritons (SPPs) resonance, propagating surface plasmon (PSP) resonance and localized surface plasmon (LSP) resonance under the excitation of TM-polarized incident light. Moreover, the Fano-like resonance mode of the designed structure can be excited under the illumination of TM-polarized oblique incident light, which is beneficial for sensing applications. Thus, it is expected that the proposed device has great potential in optical camouflage, thermal radiation, solar cells and optical sensing.

## 2. Structure and Results

**Figure 1:**
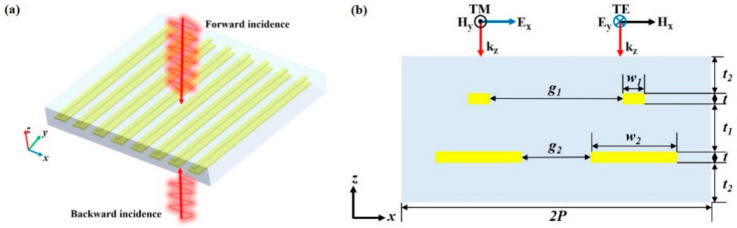
(**a**) Schematic illustration of the polarization-selective bidirectional absorber. (**b**) The double unit cells profile of the structure in the *xz*-plane.

Figure 1a illustrates the rendering of the proposed polarization-selective bidirectional perfect absorber, which consists of a one-dimensional array of bilayer gold grating buried in a silicon nitride spacer. The *xz* plane schematic indicates the geometrical parameters of the double unit cells of the designed bilayer plasmonic grating structure, as shown in Figure 1b. The period of the one-dimensional array is P = 320 nm. The width of the narrow grating is w_1_ = 45 nm, and the gap between adjacent gratings is g_1_ = 275 nm. The width of the wide grating is w_2_ = 220 nm, and the gap between neighboring gratings is g_2_ = 100 nm. The thickness of both gold gratings is t = 20 nm. The thickness of the silicon nitride spacer between the bilayer gold gratings is t_1_ = 100 nm and the thickness of the silicon nitride spacer covered onto the gold grating is t_2_ = 80 nm. Incident plane wave propagates in the *−z*- (forward) or *+z*- (backward) direction. The TM (or TE) incident wave represents the incident light with the electric field direction perpendicular (or parallel) to the grating, as shown in Figure 1b. The spectral response and field distribution of the structure are analyzed via finite element method (FEM) simulations using the commercial software COMSOL Multiphysics (Version 5.6, COMSOL Co., Ltd. 2D lujiazui financial services plaza, 1217 dongfang road, pudong new area, Shanghai, China). Periodic boundary conditions are used in the *x*-direction of a unit cell, and waveguide ports boundary conditions and perfectly matched layers are applied in the *z*-direction. The refractive index of silicon nitride is set to 2. The optical constant of gold is taken from P. B. Johnson and R. W. Christy’s work [26].

**Figure 2:**
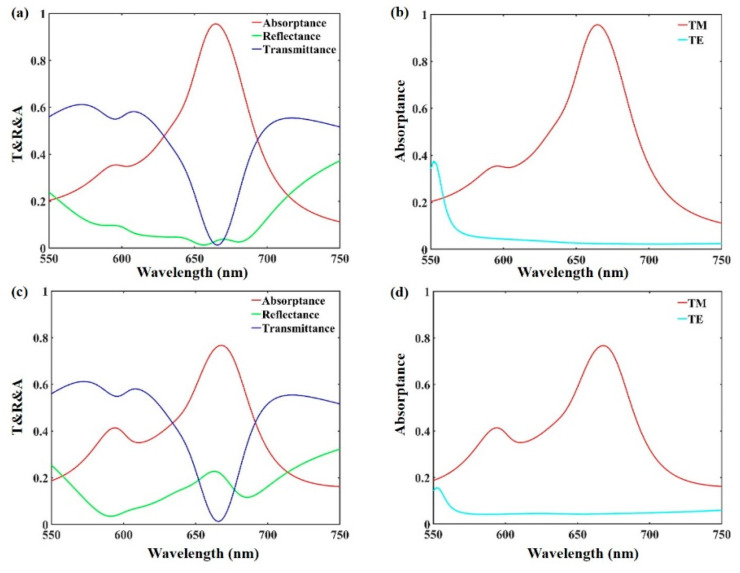
(**a**,**c**) show the absorption, transmission, and reflection spectra of the designed bilayer grating structure under forward and backward TM-polarized incidence, respectively. (**b**,**d**) show the absorption spectra of the proposed bilayer metasurface under forward and backward incident light with TE-polarized and TM-polarized irradiation, respectively.

The absorption, transmission and reflection spectra of the designed bilayer grating structure under forward TM-polarized incidence are indicated in Figure 2a. The reflection curve is near zero and an obvious decline can be observed in the transmission curve, while an absorption peak with over 95% absorptance is obtained around 666 nm. Moreover, the near-perfect absorption of incident waves in the bilayer plasmonic metasurface is polarization-selective. Figure 2b presents the absorption spectra of the proposed metasurface under both TM- and TE-polarized incidence. It can be clearly found that there is an absorption peak under TM-polarized irradiation, while the absorptance under TE-polarized irradiation is near zero around 666 nm. Similarly, we calculate the absorption, transmission and reflection spectra of the designed bilayer grating structure under backward TM-polarized incidence, as shown in Figure 2c. The obvious drop is clearly visible in the transmission, and an absorption peak with over 77% absorptance appears around 666 nm. Compared to forward incidence, the incident light first illuminates on the wide metal grating array with backward incidence, which creates a stronger reflection effect (>21%) and weakens the absorption effect of the structure. Figure 2d shows the absorption spectra under both backward TM- and TE-polarized incidence. The absorption peak only exists under TM-polarized illumination, and the absorptance is near zero under TE-polarized illumination. This means that the designed structure has the function of polarization-selective bidirectional absorption.

## 3. Analyses

**Figure 3:**
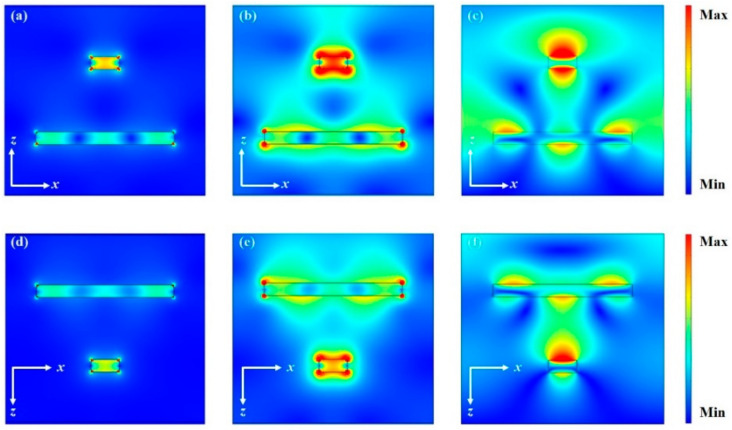
(**a**–**c**) Current density J, and electric and magnetic field distributions |E| and |H|, respectively, of the structure under forward TM-polarized light incidence. (**d**–**f**) Current density J, and electric and magnetic field distributions |E| and |H|, respectively, of the structure under backward TM-polarized light incidence.

To reveal the physical mechanism of the metasurface-based polarization-selective bidirectional absorber, the current density (J), and the electric and magnetic field distributions (|E| and |H|), of the structure under TM-polarized incidence are simulated in the *xz* plane. Figure 3 shows the field distribution of the designed structure at the resonance wavelength of 664 nm (668 nm) under normal forward (backward) incidence. As can be seen from Figure 3a, the electric current is mainly distributed in the bilayer metal gratings under the incidence of forward TM-polarized light at a wavelength of 664 nm. This means that the optical absorption loss is mainly caused by the metal grating. The electric field distribution in Figure 3b indicates that the bilayer metal gratings induce the surface plasmon polaritons (SPPs) under the excitation of incident light. The electric field localizes around the metal corners and they couple to each other in the top-layer grating due to the narrow width, which results in a strong local surface plasmon (LSP) resonance. Due to the close distance between the two gratings, light is coupled into the Si_3_N_4_ spacer between the bilayer gratings, and a weak coupling occurs with the propagating surface plasmon (PSP) resonance [27]. This can also be proven in the magnetic field distribution, as shown in Figure 3c. The magnetic field distribution is not only concentrated in the dielectric space layer above and below the metal grating, but is also significantly enhanced in the gap of the adjacent wide metal grating.

**Figure 4:**
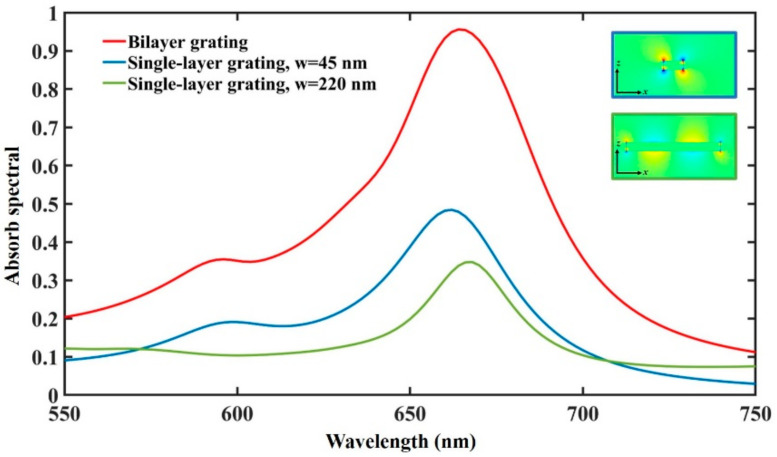
The absorption spectra of the proposed bilayer gold gratings (red line) with the narrow single-layer grating (blue line) and wide single-layer grating (green line), respectively. The blue and green boxes in the illustration represent the electric field distribution (|E_z_|) of the narrow grating and the wide grating at the resonance wavelength, respectively.

To further verify the benefits of introducing the PSP resonance, we compare the absorption spectra of the proposed bilayer gold gratings with the narrow and wide single-layer gold gratings under the forward TM-polarized incidence, respectively. The layer thickness of the single-layer grating is identical to the proposed one. The thickness of the silicon nitride spacer covered onto the single-layer gold grating is 140 nm. The absorption spectra of both cases are shown in Figure 4. We can see that the absorptivity of the bilayer grating is significantly stronger than that of the single-layer grating in the analyzed wavelength range. This is because the PSP resonance mode can be induced in the bilayer grating. The inset shows the electric field distribution (|E_z_|) of two single-layer gratings with different widths at the resonance wavelength. The blue and green boxes represent the electric field distribution of the narrow grating and the wide grating, respectively. We find that a single-layer narrow grating produces fundamental mode resonance, while wide gratings produce high-order mode resonance around 666 nm. The superposition of these two modes induces PSP mode resonance in the bilayer grating. This result also shows that the PSP resonance, SPP resonance and LSP resonance modes together lead to strong absorption at around 666 nm.

Compared to the forward incidence, the TM-polarized light excites the SPPs resonance of the wide grating under the backward incidence at a wavelength of 668 nm. Similarly, the electric current is concentrated in the metal grating, as shown in Figure 3d. Figure 3e presents that the wide grating is strongly excited by the incident wave, and there are high electric field distributions at both ends of the grating. The resulting high-order mode resonance induces the LSP resonance of the narrow grating, which is placed 100 nm away from the wide grating. The high-order mode resonance generated by wide grating array reflects part of the incident light back into the air, which results in the absorptance of the bidirectional absorber under the backward TM-polarized wave incidence being lower than that of the forward incidence. In this case, LSP resonance dominates the absorption. The magnetic field is strongly confined in the Si_3_N_4_ spacer between the bilayer gratings, as illustrated in Figure 3f. The results illustrate that the narrow grating plays an important role in the light absorption, and the resonance coupling of SPPs resonance, PSP resonance and LSP resonance produced by the bilayer grating array under the excitation of forward (or backward) TM-polarized incident light leads to strong absorption. However, the resonance coupling effect of the proposed structure cannot be acquired under the TE-polarized wave excitation (not shown here), and the bidirectional absorption cannot be obtained. Therefore, the designed double-layer grating array structure has the characteristic of polarization-selective bidirectional absorption.

## 4. Discussion

**Figure 5:**
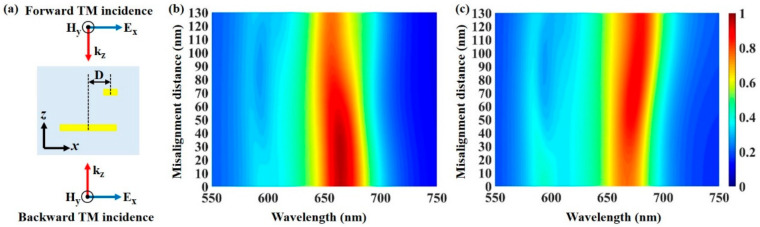
(**a**) Schematic of the center misalignment between the bilayer gratings. D indicates the center misalignment distance in the *x*-direction. (**b**,**c**) show the absorption spectra with different center misalignment distances, D, under forward and backward TM-polarized light incidence, respectively.

The previous bilayer metasurface structures have relatively high requirements for alignment accuracy, which poses a great challenge to the fabrication and cost. Here, we discuss the influence of the alignment accuracy of the bilayer gratings on the absorption effect of the designed bidirectional absorber. Figure 5a presents the *xz* plane of the device, where the narrow grating and the wide grating are not aligned, and the center misalignment distance in the *x*-direction between bilayer gratings is defined as D. The simulation results of the absorption spectra for the structure with a different center misalignment distance D under normal forward and backward TM-polarized incidence are shown in Figure 5b,c, respectively. Figure 5b shows that the absorptance keeps over 90% when the misalignment distance D is no more than 60 nm, and the absorptance keeps over 80% when the misalignment distance D is no greater than 96 nm. The absorptance even exceeds 75% when the misalignment distance D reaches 130 nm, where the center position of the narrow grating is far from the wide grating in the *x*-direction. Similarly, Figure 5c shows that the absorptance can reach more than 80% when the misalignment distance D exceeds 26 nm under the backward TM-polarized incidence. Due to the displacement increase, the magnetic field is no longer limited to the dielectric space between the bilayer gratings, and significantly increases in the gap between adjacent narrow gratings, which induces PSP resonance and enhances the optical loss. These results indicate that the designed double-layer grating structure can realize bidirectional absorption without high alignment accuracy, which greatly reduces the sample fabrication requirements.

**Figure 6:**
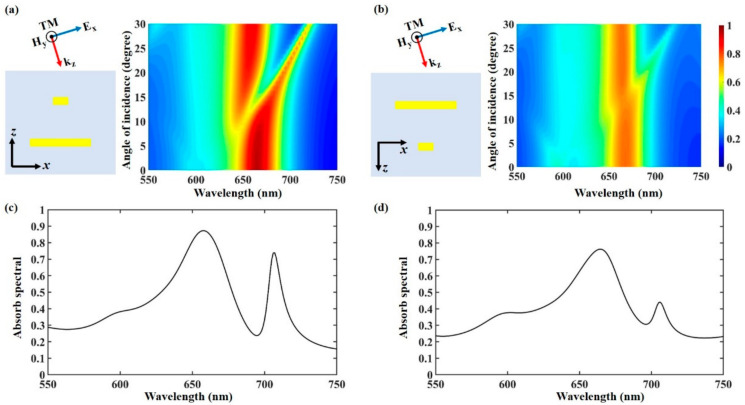
Absorption spectra of the proposed bilayer metasurface under forward (**a**) and backward (**b**) TM-polarized light incidence with different incident angles. The absorption spectral under forward (**c**) and backward (**d**) oblique TM-polarized light at the incident angle of 25°.

In addition, we investigate the influence of oblique incidence on the bidirectional absorption performance. Figure 6a indicates the simulation results of the absorption spectrum under forward TM-polarized incidence with different incident angles. It is obvious that a high absorption capability can be maintained at around 666 nm with the incident angle varying from 0 to 30°, which can be kept above 80%. A similar absorptivity can also be seen in the absorption spectrum under backward TM-polarized incidence, as shown in Figure 6b. The absorption is more than 70% within an incident angle up to 30°. Thus, the designed polarization-selective bidirectional absorber device can retain a great performance at a wide range of incident angles. It is worth noting that an absorption dip appears when the incident angle increases. The asymmetric absorption spectrum can be obtained when the incident angle is more than 9° (18°) under forward (backward) TM-polarized incidence, which shows an obvious characteristic of Fano-like resonance (an absorption rise close to an absorption peak). In order to clearly show the Fano-like resonance spectral, we plot the absorption spectral under the forward and backward oblique TM-polarized light at the incident angle of 25°, as shown in Figure 6c,d. According to the previous publications, the Fano-like resonance is induced by the interference and spatial overlap between continuous radiative resonance (bright mode) and nonradiative resonance (narrow dark mode) [28]. The Fano-like resonance in the designed structure is caused by the interference between the wide continuous spectrum resonance mode and the narrow dark resonance mode. The wide continuum resonance mode is the SPPs resonance mode of different orders on the metal grating surface, and the narrow dark resonance mode is caused by the asymmetry of the charge distribution in the metal slit. The light diffraction order is increased with the increase of the incident angle, and the symmetrical distribution of the enhanced electromagnetic field is gradually broken [29]. The left absorption peak corresponds to a continuum SPPs resonance mode with different orders on the bilayer grating, and the right narrow absorption peak is induced by the asymmetric charge distribution in the metal grating slit [30]. Therefore, the presented bilayer grating structure can maintain the function of polarization-selective bidirectional absorption within an incident angle up to 30°. The excited Fano-like resonance mode of the absorber at oblique incidence is conducive to the optical sensing application [29,30,31,32,33,34].

## 5. Conclusions

In conclusion, we have proposed a polarization-selective bidirectional absorber based on a bilayer metal grating array buried in a silicon nitride spacer. The simulation results indicate that the designed structure can realize polarization-selective absorption of TM-polarized optical waves around 666 nm. The absorption is more than 95% (77%) under forward (backward) light incidence, under normal illumination. The SPPs resonance combines with PSP resonance and LSP resonance between bilayer metal gratings, causing this great absorption effect. The calculated results also show that the misalignment distance between bilayer gratings has little effect on the absorption efficiency of the metasurface-based absorber, which significantly reduces the fabrication requirement. The absorptivity remains higher than 80% (70%) for forward (backward) TM-polarized light within an incident angle up to 30°. Moreover, the Fano-like resonance mode of the proposed metasurface can be excited with the incident angle’s increase, which is beneficial for the plasmon sensing application. Our design opens a door for the realization of polarization-selective bidirectional absorbers, which can be widely used in optical camouflage, thermal radiation solar cells and optical sensors.
